# Liquid Biopsy as a Prognostic and Theranostic Tool for the Management of Pancreatic Ductal Adenocarcinoma

**DOI:** 10.3389/fmed.2021.788869

**Published:** 2022-01-14

**Authors:** Daniel C. Osei-Bordom, Gagandeep Sachdeva, Niki Christou

**Affiliations:** ^1^Department of General Surgery, Queen Elizabeth Hospital, University Hospitals Birmingham, Birmingham, United Kingdom; ^2^Institute of Immunology and Immunotherapy, University of Birmingham, Birmingham, United Kingdom; ^3^National Institute for Health Research (NIHR) Birmingham Biomedical Research Centre, Centre for Liver and Gastroenterology Research, University of Birmingham, Birmingham, United Kingdom; ^4^Department of General Surgery, University Hospital of Limoges, Limoges, France; ^5^EA3842 CAPTuR Laboratory “Cell Activation Control, Tumor Progression and Therapeutic Resistance”, Faculty of Medicine, Limoges, France

**Keywords:** pancreatic ductal adenocarcinoma, liquid biopsy, exosomes, prognosis, personalized treatment, circulating free DNA (cfDNA), circulating free microRNAs

## Abstract

Pancreatic ductal adenocarcinomas (PDAC) represent one of the deadliest cancers worldwide. Survival is still low due to diagnosis at an advanced stage and resistance to treatment. Herein, we review the main types of liquid biopsy able to help in both prognosis and adaptation of treatments.

## Introduction

Biopsies represent a fundamental tool for clinical assessment, aiding with the diagnosis and management of the disease. In the oncological setting, tissue biopsy with fragments of primary/metastatic tumors has been traditionally used for the histological classification of disease and, more recently, for the genetic mutational profiling of cancer. However, evolving techniques for the analysis of histological samples have highlighted the limitations associated with such assessment (1). In addition, concerns over single-biopsy bias have been raised, with Gerlinger et al. demonstrating intertumoral and intratumoral heterogeneity between different sites on the primary tumor and its metastasis (2). Therefore, single-biopsy risks underestimating the complexity of the histological and genomic landscape of the tumor and serial monitoring with repeated tissue biopsies does not prove easily feasible. These limitations have highlighted the need for a minimally invasive approach for the systematic and real-time monitoring of cancer. In the recent years, the minimally-invasive liquid biopsy technique has emerged, utilizing body effluents (mainly blood) to identify cancer biomarkers, and assess tumor biochemistry and genetic status on a systemic level (3–5). Types and categories of liquid biopsy can be classified by the type of material analyzed and the type of analysis to be performed (6). A significant positive with respect to using liquid biopsies rather than tissue biopsies is that we can capture all the genetic material released from all the tumor sites, thereby enabling us to portray whole tumor heterogeneity (7).

**Figure 1:**
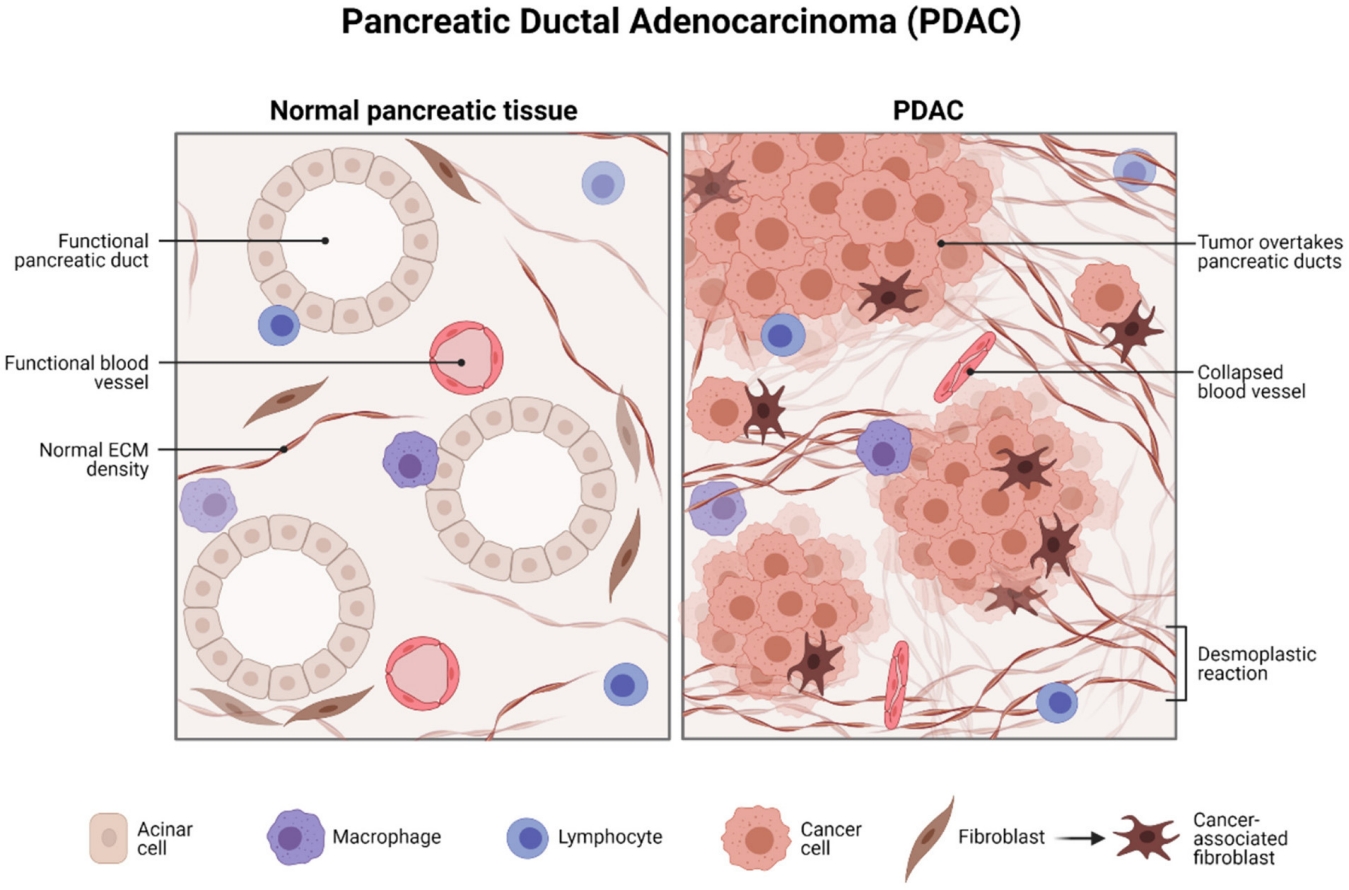
Histology of pancreatic ductal adenocarcinoma. Created with BioRender.com.

Pancreatic ductal adenocarcinoma (PDAC) represents one of the deadliest cancers worldwide. Survival is still low due to diagnosis at an advanced stage and resistance to treatment. There is a drive to identify biomarker/s that facilitates in the diagnosis, prognosis, and development of personalized treatment strategies. This study will focus on the potential role of liquid biopsy as a prognostic and theranostic tool for managing PDAC (Figure 1).

## Pancreatic Ductal Adenocarcinoma: Current Methods of Diagnosis and Their Challenges

Pancreatic ductal adenocarcinoma represents the most common type of pancreatic cancer and one of the most aggressive solid tumor malignancies. PDAC poses a significant challenge. Given its typical silent course and late presentation by which the tumor is mainly unresectable, 1-year overall survival (OS) and 5-year OS are 24 and 5% (or less), respectively (8, 9). Epidemiological data suggest that PDAC is the 7th leading cause of global cancer-related deaths and 3rd leading cause in the United States (US) (10, 11). Furthermore, by 2030, it is predicted that PDAC will be the 2nd most common cause of cancer deaths, preceded by lung cancer (12).

Aside from asymptomatic disease, the clinical presentation of PDAC may include weight loss, abdominal pain, jaundice, steatorrhea (10, 13), and less commonly type 2 diabetes mellitus (14) and thromboembolic disease (15). In addition, patients may develop pancreatic exocrine insufficiency (PEI), secondary to tumor-driven obstructive damage to the secreting component of the pancreas (16).

Guidelines dictate that the preferential investigation to aid with diagnosis in patients with suspected pancreatic cancer is a pancreatic protocol CT, with secondary fluorodeoxyglucose (FDG)-PET/CT and endoscopic ultrasound (EUS)-guided tissue sampling should the diagnosis remain unclear (17). Surveillance for pancreatic cancer should be considered in patients with an inherited high risk: family history, hereditary pancreatitis, and genetic mutations [Serine Protease 1 (PRSS1), breast cancer gene 1/2 (BRCA1/2), partner and localizer of BRCA2 (PALB2), cyclin dependent kinase inhibitor 2A (CDKN2A), mutl homolog 1 (MLH1), muts homolog 2/6 (MSH2/6), PMS1 Homolog 2, Mismatch Repair System Component (PMS2)], by way of magnetic resonance imaging (MRI) magnetic resonance cholangiopancreatography (MRCP) or EUS (17). Upon confirmation of the diagnosis of pancreatic cancer, staging should be performed, including FDG-PET/CT in patients with the localized disease on CT when planning for surgery, radiotherapy, or systemic therapy. MRI, EUS, and laparoscopy with laparoscopic US may also be performed to support decisions for management (17).

Endoscopic US-guided tissue sampling is an invasive surgical approach to cytological and histological analysis of tumors and does not overcome concerns over a single biopsy bias. However, given an appropriate efficacy study, liquid biopsy provides the scope for minimally invasive sampling as a systemic level diagnostic tool for tumor analysis.

## Management of PDAC and Methods of Surveillance

Pancreatic ductal adenocarcinoma exhibits rapid tumor progression, with limited efficacy of current therapeutic drug regimes in locoregional and metastatic disease (18). In resectable and borderline resectable cases of PDAC, mainstay treatment remains surgical resection in combination with chemoradiotherapy; however, by the time of clinical presentation, the advanced tumor stage renders over 80% of cases as unresectable (19, 20). Metal stenting to relieve biliary and duodenal obstructions may be performed in patients not suitable for resection (unresectable or not fit for surgery). Gemcitabine-based chemotherapy and combination chemotherapy, including folfirinox (irinotecan, oxaliplatin, 5-fluorouracil, and leucovorin), demonstrate a moderate benefit for OS (21). Novel immunotherapeutics for PDAC prove promising for targeted therapy, with pembrolizumab as an the United States Food and Drug Administration (FDA)-approved programmed cell death protein 1 (PD-1) checkpoint inhibitor for unresectable and metastatic mismatch repair-deficient/microsatellite instability-high (dMMR/MSI-H) solid tumors (22) and other agents under investigation in clinical trials (23–28). Nutritional support with replacement therapy, pain management, and psychological support also proves essential for holistic patient care.

Surveillance during the management of PDAC and after curative surgery also plays a role in the risk stratification and identifying prognostic factors in patients. First, baseline performance status (PS) of patient has been recognized as an essential clinical biomarker and independent prognostic factor for treatment response. Poor baseline has been negatively correlated with chemotherapeutic response rates, including gemcitabine, and less favorable clinical outcomes (29, 30). Second, biological prognostic biomarkers in PDAC have also been suggested, including carbohydrate antigen 19-9 (CA 19-9) and activin A. Elevated baseline CA 19-9 (pancreatic tumor marker) and activin A (transforming growth factor beta (TGF-β) superfamily cytokine) have been correlated with worse prognosis, with a role in postoperative recurrence and metastatic spread, respectively (31, 32). Results from an early meta-analysis that looked at the specificity and sensitivity of the use of common serum biomarkers highlighted that for carcinoembryonic antigen (CEA) and CA 19-9. The mean sensitivity and specificity estimates for CEA were 44.2 and 84.8%, respectively, and for CA 19-9, the mean sensitivity and specificity were 78.2 and 82.8%, respectively. The analysis included numerous studies that include patients with benign pancreatic disease including chronic pancreatitis and cholelithiasis (33). Due to a range in sensitivities and specificities, the importance of having markers to monitor patient outcomes for treatment planning. An intensified chemotherapy regimen has been advised in patients with elevated CA 19-9 (31) and activin A neutralizing antibodies have been studied for decreasing tumor metastasis (32).

## Use of Liquid Biopsies in the Detection and Surveillance in PDAC

First, with respect to the type of material analyzed, tumor-released markers that may be detected in liquid biopsy include circulating tumor cells (CTCs), circulating free nucleic acids [circulating free DNA (cfDNA), circulating free RNA (cfRNA), circulating free microRNA (cfmiRNA)], exosomes, and tumor-educated platelets (TEPs) (6, 34, 35).

Circulating tumor cells are among the most extensively studied markers and represent cancer cells that have gained plasticity and motility by epithelial–mesenchymal transition (EMT), extravasating from the tumor primary and into systemic circulation (35, 36). Principally, CTCs can lead to cancer metastasis through intravasation into distant tissue and, therefore, prove critical in cancer diagnostics and prognostics. In the context of cancer, cfDNA, cfRNA, and cfmiRNA are nucleic acid fragments discharged into circulation from apoptotic cells and necrotic tumor cells (35). Important to recognize is that most cfDNA is non-malignant in origin. However, when cfDNA derives from tumor cells, it is called circulating tumor DNA (ctDNA), carrying tumor-specific mutations (37); there is evidence suggesting elevated levels of cfDNA in the later stages of cancer (38). Circulating free nucleic acids are unstable in blood and other sources for genetic analysis include circulating microvesicles (exosomes) and TEPs (35, 39, 40).

Liquid biopsy can also be categorized utilizing the type of analysis including small- and large-scales mutation analysis, with targeted deep sequencing and next-generation sequencing, and analysis of structural change and copy number alterations (6). Mutational profiling recognizes point mutations, insertions, deletions, and genomic strands, which may aid with the metric classification of prognosis, resistance, disease burden, and therapeutic efficacy of medicines (41–43). Structural analysis using the chain termination method (Sanger method) involves the identification of single nucleotide polymorphisms (SNPs), structural and copy number variations, and focal mutational profiling as above (6, 44). cfDNA can also be studied to identify copy number alterations (45). Thus, liquid biopsy appears promising and demonstrates suitability for application into conventional cancer care (46–49).

## Circulating Tumor Cells in PDAC

The concept of the liquid biopsy in PDAC is to ultimately paint a molecular genetic landscape of PDAC, which will improve the characterization of PDAC heterogeneity, aid in the early detection and treatment surveillance, with minimal circulating tumor material (50). CTCs and circulating tumor-derived proteins have been used clinically including CA 19-9 and prostate-specific antigen (PSA) used for detecting and surveillance pancreatic cancer and prostate cancer, respectively. There is hope that by utilizing CTCs or circulating tumor-derived proteins, we would be able to develop a marker test that is highly sensitive and specific in detecting PDAC (51, 52).

**Figure 2:**
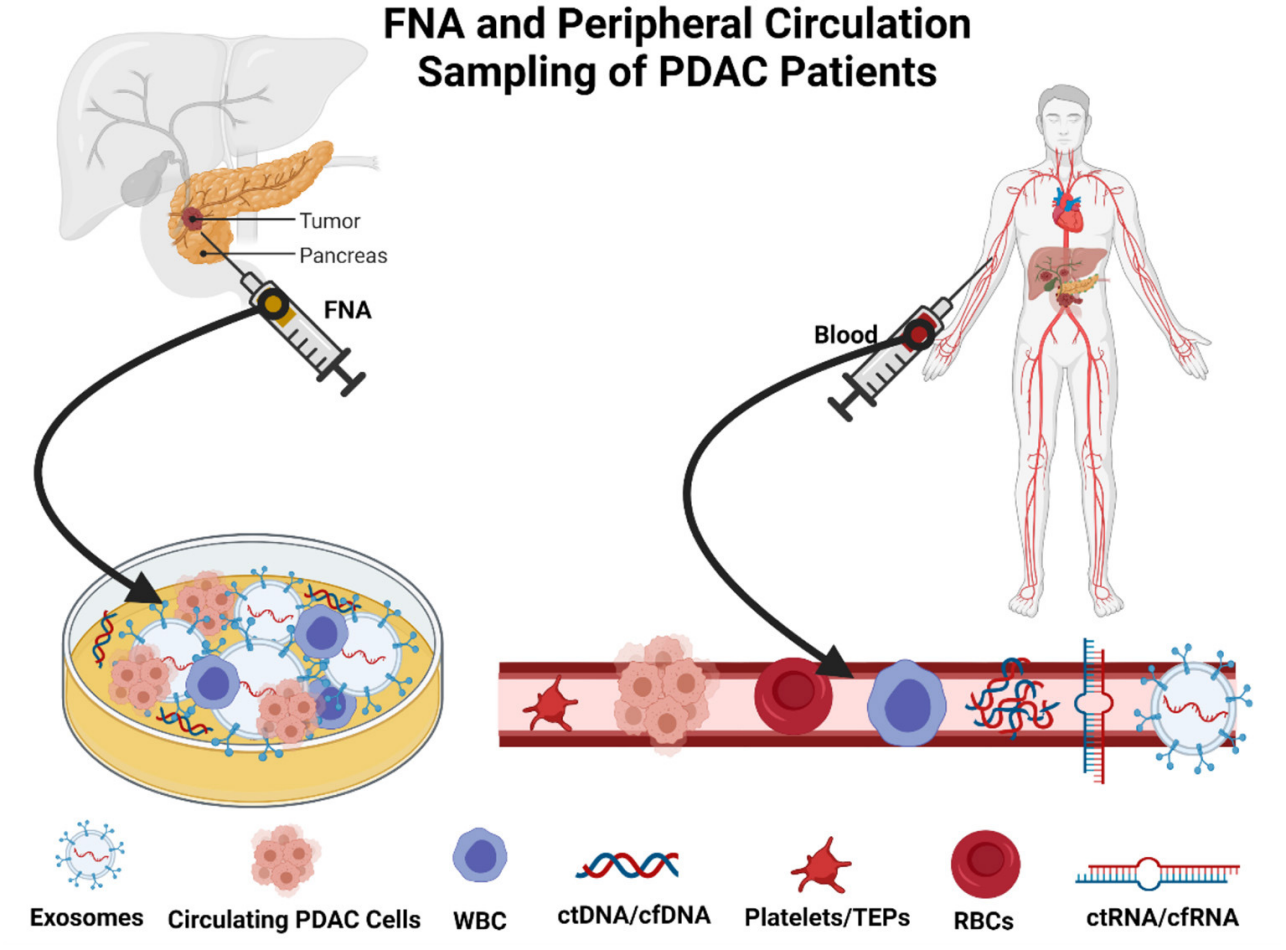
Method of sampling in patients with pancreatic ductal adenocarcinoma (PDAC). Created with BioRender.com.

The methodology relies on the use of low volume micromaterials released from the PDAC, which releases components, including cell-free DNA (cfDNA), cell-free RNA (cfRNA), extracellular vesicles (EVs), and CTCs, into bodily fluids including blood, fine needle aspirates at biopsy, and saliva (53, 54). CTCs comprise a low percentage of circulating material released from the PDAC tumors. However, despite their small collective proportion of circulating tumor biomaterial, it is also found in clusters and can be found as single cells (55). Singular cells are often detected in the peripheral circulation and can be seen in blood biopsies (Figure 2). There are relative obstacles in using CTCs as a biomarker for early detection and surveillance of PDAC (54, 56). First, there is a fact that the presence of CTCs does not always correlate to the presence of malignant invasive PDAC and, as aforementioned, the low yield of CTCs in peripheral circulation; the theoretical reasoning for the low yield of CTCs is primarily due to their relative size mainly among other factors (57, 58). CTCs are often 3–5 times the size of capillary openings before entering central circulation via the portal vein. Therefore, the larger CTCs remain trapped either within the capillaries and the smaller CTCs enter peripheral circulation (59–61). The anatomical location and blood drainage that supplies the pancreas will directly drain into the portal vein via the splenic vein. This further implicates the choice of attaining peripheral CTCs and whether the use of peripheral blood and presents portal vein sampling as a preferential option for point of sampling to allow for early diagnosis (62, 63), disease stratification, and longitudinal monitoring of therapeutic response in patients with pancreatic. CTCs are exceedingly heterogeneous concerning PDAC tumor biology (62, 64, 65), which is often a benefit compared to a singular tumor biopsy. Several techniques are described to isolate CTCs and their derived proteins including mass spectrometry and proteomics. Antibody-affinity binding-based technology has been documented as highly specific and sensitive in detecting novel biomarkers from liquid biopsies (66, 67). For PDAC, in the context of utilizing CTCs and other components of a liquid biopsy are compared with the diagnostic marker, CA19-9 (68). Proteins of interest from liquid biopsies are glypican 1 (GPC1), epithelial cellular adhesion molecule (EpCAM), CD45, CD69, tissue inhibitor of metalloproteinase 1 (TIMP1), thrombospondin-2 (THBS2), leucine-rich alpha-2-glycoprotein 1 (LRG1) and also, more importantly, kirsten rat sarcoma viral oncogene homolog (KRAS) mutations (69, 70). Blood metabolomics has also been indicated as a method of detecting early stages of PDAC and surveillance of the recurrence of PDAC. Recent investigations highlight that the plasma metabolites such as acetylspermidine, diacetylspermine, indole derivatives, and lysophosphatidylcholines could distinguish PDAC from healthy subjects benign pancreatic disorders (68, 71).

Often, the metabolites mentioned are combined with tumor associated proteins of PDAC, including CA 19-9, to monitor early detection of PDAC and tumor progression. The highly diagnostic and prognostic value of using CTCs from liquid biopsies, particularly from solid gastrointestinal tumors, is primarily due to CTCs from direct natural shedding of CTCs and detachment from a single or multiple loci of the primary PDAC tumor as well as metastases into the peripheral circulation and often are the primary sources of metastases (72, 73). The immunobiology of CTCs captures the whole-body burden of tumor than single point tissue biopsies; they highlight the tumor heterogeneity. The favoring of CTCs rather than exosomes, ctDNA, cfDNA, ctRNA, or cfRNA or any other components of liquid biopsies due to its stored biogenetic information encodes for various expressive influential genes and proteins, particular in the development of a complex, comprehensive landscape of tumor heterogeneity and evolution (74, 75). Thus, CTCs play an integral role in testing current and new therapeutics; the additional benefit of using CTCs includes the tumor heterogeneity, which can be developed into an *ex-vivo* culture that genetically mirrors the whole PDAC tumor (76, 77). This allows clinicians to assess personalized therapeutic regimes and identify and predict the success of therapeutic regimes tested and potential new immunotherapies developed (78). Processes including reverse transcription-PCR (RT-PCR) and ultra/standard density centrifugation have been used to isolate CTCs of PDAC tumor cells from whole blood based on CEA, cytokeratin-20 (CK-20), and EpCAM (79–81) (Table 1).

**Table 1:** Sampling materials and methods of clinical application with PDAC samples.

**Type of materials**	**Diagnostic detection**	**Method of detection**	**References**
ctDNA/ctRNA	Amplification, deletion and translocation Gene driver mutations, Chromosomal abnormalities	PCR Whole genome sequencing Next-generation sequencing	Fabbri et al. (82)
CTCs	Gene expression Cell Proliferation/growth Cell differentiation Cell mobility Apoptosis	Next-generation sequencing Microarray testing qRT-PCR CTC Chip sequencing EPISOT Phenotypic analysis: immunostaining, FACS and LC-MS	Pantel et al. (37)
miRNA	Gene fusion transcripts RNA expression	Next-generation sequencing Microarray testing qRT-PCR	Tesfaye et al. (83)
TEPs	Gene expression	Next-generation sequencing RNA sequencing	Best et al. (84) Joosse et al. (40)

*PDAC, pancreatic ductal adenocarcinoma; LC-MS, liquid chromatography-mass spectrometry; FACS, fluorescence-activated single cell sorting; qRT-PCR, real time quantitative reverse transcription polymerase chain reaction*.

## Circulating Tumor DNA and RNA

### Circulating Tumor DNA

Circulating tumor DNA of PDACs is representative of a short genomic fragment coding for the PDAC tumor spanning all the chromosomes. ctDNA is found in peripheral circulation, which often originates from the necrosis or apoptosis of the primary PDAC tumor or from metastatic PDAC (85). ctDNA also encompasses a proportion of circulating cfDNA from PDAC tumor cells. The general base pair size of circulating ctDNA is usually about 150–160 base pairs; often, these pairs are associated with nucleosomes, contributing to ctDNA detection (86). In the evolving epigenetic landscapes of pancreatic cancer, the ability to access the DNA of PDAC tumors is imperative to orchestrate a personalized precision therapeutic regime (87). Nucleosomes facilitate access to DNA due to several metabolic reactions. These remodeled nucleosomes move along the DNA and expose or cover the base regions in particular transcription factor binding sites. In a typical physiological environment, these nucleosomes interact with and unify many nuclear components and processes required for gene expression (88, 89). Remarkably, there are mutations present in multiple of the components that facilitate the remodeling of nucleosomes that have also been explicitly found in PDAC, including members of the switch/sucrose non-fermentable (SWI/SNF) family: brahma-related gene-1 (BRG1), SWI/SNF Related, Matrix Associated, Actin Dependent Regulator Of Chromatin, Subfamily A, Member 4 (SMARCA4), BRG-associated factor 250a (BAF250a (ARID1A)) or BRG-associated factor 250b (BAF250b (ARID1B)) and brahma homolog (BRM (SMARCA2)) among others, suggesting a crucial role for nucleosome positioning in the establishing PDAC cancer phenotype and heterogeneity (90–92). The exposed non-coding regions of RNA or DNA, controlled by the nucleosomes, have a direct impact on the epigenetics of PDAC. Due to their prevalence as circulating exosomes encapsulated in body fluids, these exosomes and nucleosomes offer potential therapeutic targets as well as biomarkers for diagnosis and monitoring disease progression (93, 94).

Circulating tumor DNA presents as precious markers particularly for being: (1) a biomarker of PDAC for early detection of the onset of PDAC and surveillance of PDAC recurrence and (2) a biomarker, which facilitates decision-making to permit the most appropriate therapeutic regime (95, 96). Aforementioned, including the critical roles in PDAC detection and treatment, ctDNA has been highlighted in several studies to correlate to PDAC tumor burden, primarily more sensitive in detecting advanced PDAC cancer detection than early-stage PDAC malignancy (97). Furthermore, studies have highlighted that plasma KRAS and epidermal growth factor receptor (EGFR) mutations act as the most predictive and reliable biomarker for detecting and surveillance PDAC, particularly KRAS G12D mutation (98, 99). Reportedly, there is a significant association between the KRAS G12D mutation and the appearance with “micrometastasis” within patients with PDAC, thereby allowing clinicians to stratify high-risk and low-risk patients preoperatively and guide appropriate initial treatment regimes (100).

With respect to ctDNA, there are a number of widely used applications in the identification and quantification technique of ctDNA. For example, identifying EGFR-tyrosine kinase (PTK) mutations from ctDNA for diagnostic purposes in patients with non-small cell lung cancer (NSCLC) (101, 102). The identification of mutations in the EGFR-PTK pathways and testing potential therapeutics involving EGFR-PTK inhibitors and highlighting patients who are specifically sensitized or resistant to this treatment method. ctDNA has also been used in assisting in the detection and recurrence surveillance for colorectal and breast cancers. Utilizing ctDNA monitoring in these cancer types has been extremely promising in highlighting appropriate patients for particular treatment pathways and identify specific therapeutic regimes that will have the most clinical benefit (103, 104).

### Circulating Tumor RNA

Circulating tumor RNA is also included in the group of important circulating biomarkers extracted from liquid biopsies. Among the essential circulating biomarkers in peripheral blood include ctRNAs mentioned above, but subclassification of ctRNAs also includes: miRNAs, TEPs, and metabolites (105–107). The importance of ctRNA is that they express particular non-coding regions of genetic transcripts of PDAC tumors. The significance of ctRNA is implicated when investigating the RNA expression profiles of extracted components of the liquid biopsy (108). Studies have shown that the RNA profiles of TEPs have been shown to discriminate between being in a patient with PDAC tumors compared to TEPs in circulation in the presence of healthy tissues. This is primarily due to platelets RNA biomarker signatures altering by the presence of cancer (109, 110). Microarray studies investigating platelets from the control group of inflammatory diseases identified 22 differentially expressed genes. The expression levels of these genes are increased in patients with cancer (84). Platelet markers that alter significantly in the presence of cancer have been identified as beta-2-microglobulin (B2M), pro-platelet basic protein (PPBP), thymosin beta 4 X-Linked (TMSB4X), and platelet factor 4 (PF4) (111). Due to their molecular uniqueness in the presence of cancers, TEPs can also subclassify multiple types of gastrointestinal solid cancers (107). MicroRNA (miR) identification has a prominent role in PDAC detection, monitoring, and surveillance.

Several significant circulating miR molecular signatures have been important in the early detection and surveillance of PDAC (112). Several studies highlight the miRs: miR-17-5p, miR-21, miR-155, miR-196a, miR-17-5p, and miR-21. All of which have been shown to have a considerably high diagnostic sensitivity and specificity (113, 114). When investigating and treating patients at early stages of PDAC and treating patients with premalignant pancreatic lesions, including mucinous cystic neoplasms (MCNs) and intraductal papillary mucosal neoplasms (IPMNs), miRs play a pivotal role in indicating a malignant transformation and initiating the appropriate treatment at the correct timepoint (115). When analyzing the expression of miR-191, miR-451a, and miR-21 in pancreatic cancer and IPMNs, we see that the expression levels dramatically increased in all the three miRs when comparing the IPMN cohort vs. the PDAC cohort (114, 116). These miRs have be shown to have a greater diagnostic process when compared to the conventional clinical markers including CA-19.9 when monitoring the transformation of premalignant lesion of IPMN to cancerous early-/late-stage PDAC (117).

## Circulating EVs

Extracellular vesicles are essentially circulating nano-sized proportions containing nucleoprotein material products of cell types they have originated from, more importantly in this instance from tumor cells including from PDAC (118, 119). These vesicles are formed by distinct loading mechanisms that encapsulate RNA and protein profiles from origin parent cells, thus highlighting a sophisticated and selective loading mechanism employed. Hence, EV has gained significant interest and is highly sought after as potential diagnostic biomarkers. The genetically significant proteins that EV hold often facilitate the mapping and interpretation and the genetic and metabolic landscape of the tumor stem from (120). EVs have a considerably longer half-life when compared to ctDNA, ctRNA, or circulating miR. They are highly abundant in peripheral circulation due to their consistent production from PDAC tumors, irrespective of the rates of tumor cell necrosis, apoptosis, or invasion (121). These factors impact their use as markers for early detection and increase the ability to be yielded from a number of sources including ascites, blood, and saliva (122, 123). Particularly in regards to PDAC samples and attaining a traditional fine needle aspiration (FNA) biopsy can be problematic often yields low volume and low-quality tissue for conventional tissue diagnosis protocol (124). However, using EVs from these FNA biopsies samples, clinicians can explore protein and genetic analysis for necessary diagnostics for specifically targeted therapy (125).

MicroRNA has been highlighted as a potential key identifier for PDAC. The level of expression of their genetic signature theoretically correlates to improved sensitivity and specificity of microarrays for detecting PDAC (126, 127). In reference to future directions of the use of exosomes, EV and CTCs, for clinical activity, are that we need to develop a means in which we can increase the rate of sensitivity and specificity in regard to accuracy of testing and selection of genetic materials including nucleic acids and proteins to unwind the heterogeneous genomic picture of the PDAC tumors (128).

## Conclusion

The availability of liquid biopsy has allowed clinicians to study peripheral blood and FNA biopsy samples of patients with PDAC for clinical applications including early detection and surveillance, to optimize overall survival outcomes in affected patients. Exosomes, EVs CTC, ctRNA, and ctDNA have been investigated to provide a matching analysis of PDAC tumor genomic profiles confirming heterogeneity; this finding is essential in highlighting potential therapeutic targets against PDAC variants personalizing precision medicine for each patient afflicted by PDAC. The clinical relevance of liquid biopsies and genomic profiling of PDAC has not only been used for the monitoring of early-to-late PDAC and surveillance of recurrence, but the sampling of the genetic material of exosomes, EVs CTC, ctRNA, and ctDNA assists with the treatment monitoring and for prognostic purposes in patients. Large-scale retrospective and prospective studies are required to rigorously assess the validity of the use of liquid biopsies safely in the clinical environment to manage PDAC in patients.

## Author Contributions

DO-B, GS, and NC: conceptualization, methodology, software, validation, formal analysis, investigation, resources, data curation, writing—original draft preparation, writing—review and editing, and visualization. All authors contributed to the article and approved the submitted version.

## Conflict of Interest

The authors declare that the research was conducted in the absence of any commercial or financial relationships that could be construed as a potential conflict of interest.

## Publisher's Note

All claims expressed in this article are solely those of the authors and do not necessarily represent those of their affiliated organizations, or those of the publisher, the editors and the reviewers. Any product that may be evaluated in this article, or claim that may be made by its manufacturer, is not guaranteed or endorsed by the publisher.
